# Case Report: Hereditary transthyretin (ATTRv) amyloidosis: The p.G103R mutation of the transthyretin gene in a Han Chinese family is associated with vitreous hemorrhage

**DOI:** 10.3389/fgene.2022.972501

**Published:** 2022-09-15

**Authors:** Junhui Shen, Hao Yu, Jijian Lin, Li Zhang, Xiaohong Pan, Zhiqing Chen

**Affiliations:** ^1^ Eye Center, Second Affiliated Hospital, Zhejiang University School of Medicine, Hangzhou, China; ^2^ Department of Neurology and Department of Medical Genetics in Second Affiliated Hospital, and Key Laboratory of Medical Neurobiology of Zhejiang Province, Zhejiang University School of Medicine, Hangzhou, China; ^3^ Department of Cardiology, Second Affiliated Hospital, Zhejiang University School of Medicine, Hangzhou, China

**Keywords:** hereditary transthyretin (ATTRv) amyloidosis, transthyretin gene, mutation, vitreous opacity, vitreous hemorrhage

## Abstract

Hereditary transthyretin (ATTRv) amyloidosis is a rare disease caused by transthyretin gene (*TTR*) mutation. We identified that the p.G103R mutation of the *TTR* gene in a Han Chinese family was associated with vitreous hemorrhage. The proband was a 48-year-old woman who had progressive visual impairment in both eyes for 12 years. A Glass wool–like posterior vitreous cortex attached to the posterior retinal surface of both eyes was found using ocular coherence tomography. Visual acuity improved after the first vitrectomy. Two years later, the patient underwent two more vitrectomies because of vitreous opacity recrudescence. Four years later, she presented with vitreous hemorrhage in the right eye. The vitreous fluids acquired during the vitrectomy showed increased vascular endothelial growth factor, basic fibroblast growth factor, interleukin-6, interleukin-10, vascular cell adhesion molecule, and interleukin-8. Mutation sequencing revealed a heterozygous mutation in nucleotide c.307G > C (p.G103R) in exon 3 of the *TTR* gene in the proband (IV-13), her daughter (IV-9), and her fourth sister (III-11). To our knowledge, this is the first case of ATTRv amyloidosis caused by a p.G103R mutation of the *TTR* gene associated with vitreous hemorrhage in China.

## Introduction

Amyloidosis is characterized by the abnormal accumulation of insoluble amyloid fibril in the extracellular matrix. Amyloidosis can be divided into systemic and localized amyloidosis according to the affected sites (Casal et al., 2016). The common organs involved are the kidneys, heart, skin, and eye. Ocular amyloidosis is mainly due to the accumulation of amyloid fibril in the vitreous, which leads to visual impairment (Obici et al., 2021). It either can be the first and/or only clinical feature, or the ocular manifestation of systemic amyloidosis. The ocular manifestations include the deposition of amyloid proteins in the lens and the margin of the pupil, abnormal conjunctival vessels, glaucoma, vitreous opacity, and retinal amyloid angiopathy (Bezerra et al., 2019).

It has been reported that several genes are pathogenic factors of this disease. The transthyretin (*TTR*) gene is the main genetic factor, and 99% of cases are due to mutations of this gene. *TTR* can transport thyroid hormones and vitamin A. Presently, there are at least 13 *TTR* gene mutations related to ocular amyloidosis in China, the most common of which include p.R54G, p.K55T, p.G67E, p.L75R, and p.G103R (Yining et al., 2011; Long et al., 2012; Zhang et al., 2013; Leung and Ko, 2019). Ocular amyloidosis is a rare disease in China. Only 39 families with this disease have been reported (Leung and Ko, 2019). This study described an ocular amyloidosis patient with a p.G103R mutation in *TTR* who presented with vitreous hemorrhage.

## Results

### Case presentation

**FIGURE 1:**
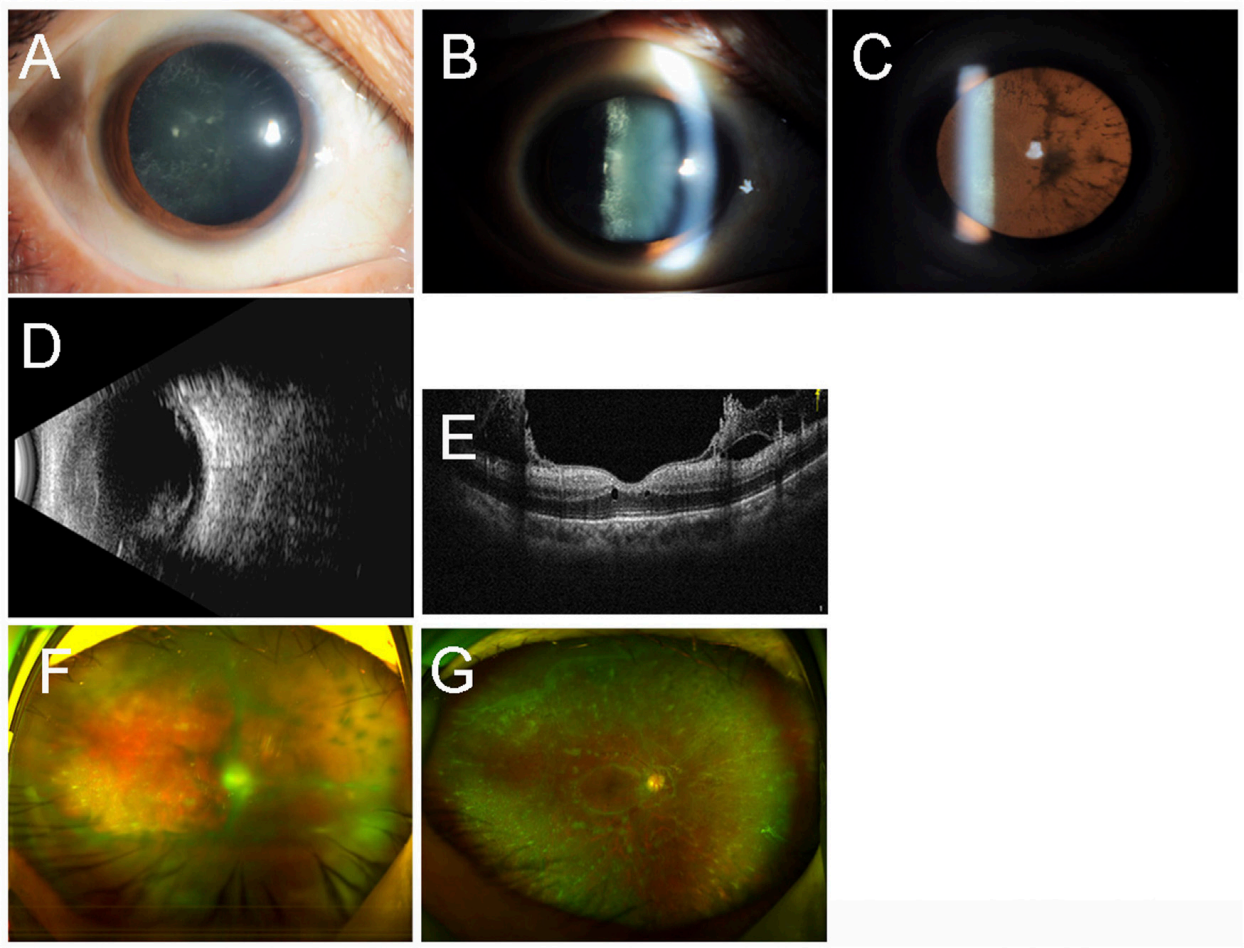
Ocular imaging findings of the right eye of the proband (III-13). Slit-lamp examination **(A–C)** revealed amyloid deposition in the posterior lens capsule. B-mode ultrasonography **(B)** showed mass echoes in the vitreous cavity. Ocular coherence tomography **(E)** confirmed the glasswool-like posterior vitreous cortex attached to the retina. Ultra-wide-field scanning laser ophthalmoscopy revealed vitreous opacities before the surgery **(F)** and one month after the surgery **(G)**.

The proband (III-13) was a 48-year-old woman who had progressive visual impairment in both eyes for 12 years. Her best-corrected visual acuity (BCVA) was 0.2 in the right eye and 0.4 in the left eye. Her intraocular pressure (IOP) was 31 mmHg of the right eye and 13 mmHg of the left eye. Amyloid deposition was found in the posterior lens capsule of the right eye by slit-lamp examination (Figures 1A–C). Mass echoes were found in the vitreous cavity by B-mode ultrasonography of the right eye (Figure 1D). A glass wool–like posterior vitreous cortex attached to the posterior retinal surface of the right eye was found using ocular coherence tomography (Figure 1E). Ultrawide-field scanning laser ophthalmoscopy (SLO) revealed vitreous opacities in the right eye (Figure 1F). The patient underwent cataract extraction, intraocular lens implantation, pars plana vitrectomy (PPV), and gas tamponade in her right eye. Multiple opacities could be seen near the peripheral retinal vessels that did not involve the macular area in the right eye one month after surgery (Figure 1G). The BCVA in the right eye was 0.5 after surgery.

Then, the patient complained of decreased vision in the left eye. During ophthalmologic examination, fundus examination showed a large number of yellow-white, lumpy, cotton wool–like deposits in the vitreous bodies of both eyes. She underwent PPV surgery in another hospital for her left eye because of visual impairment and vitreous opacity. The BCVA in the left eye was 0.8 after the surgery. Two years later, she underwent two more vitrectomies in her right eye at other hospitals.

**FIGURE 2:**
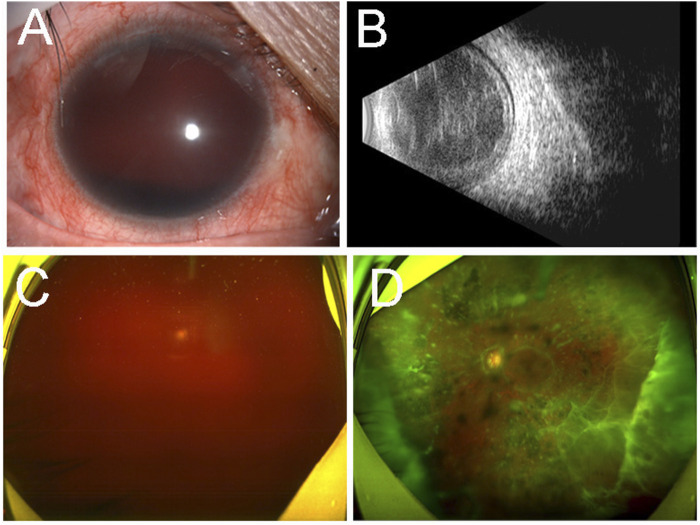
Ocular imaging findings of the both eyes of the proband (III-13) four years later. Slit-lamp examination **(A)** revealed hyphema of the right eye. B-mode ultrasonography **(B)** showed mass echoes in the vitreous cavity. Ultra-wide-field scanning laser ophthalmoscopy revealed vitreous hemorrhage in the right eye **(C)** and multiple flocculent degenerations in the vitreous of the left eye **(D)**.

Four years later, she was referred to ophthalmology due to blurred vision in her right eye for six months. Her BCVA was no light perception in the right eye and 0.6 in the left eye. Her IOP was 28 mmHg of the right eye and 14 mmHg of the left eye. Hyphema of the right eye was found by slit-lamp examination (Figure 2A). B-mode ultrasonography showed mass echoes in the vitreous cavity (Figure 2B). The SLO revealed vitreous hemorrhage in the right eye (Figure 2C). Multiple flocculent degenerations in the vitreous could be seen in the left eye with SLO (Figure 2D).

Acetazolamide was given to lower the patient’s IOP, which then returned to normal. She underwent PPV and silicone oil tamponade in her right eye. During the operation, retinal vein occlusion, retinal edema, and large deposits of amyloid in the anterior and subretinal areas were observed. The vitreous body adhered closely to the retina to form traction hyperplasia, leading to secondary retinal detachment. Retinal holes in the peripheral retina were also found. She received an intravitreal injection of an anti-VEGF agent after the surgery. The vitreous fluids acquired during the surgery were sent for cytokine analysis (Flow Cytometry Analysis, Beijing Giantmed Medical Diagnostics Lab). Notably, VEGF (192.6 pg/ml), BFGF (18.2 pg/ml), IL-6 (21,045.1 pg/ml), IL-10 (14 pg/ml), VCAM (35,390.8 pg/ml), and IL-8 (4,361.8 pg/ml) concentrations had increased significantly. Intraocular pressure was normal after the surgery; unfortunately, she experienced vitreous hemorrhage one day after the surgery.

The patient did not have any neurologic complaints, such as limb numbness or weakness. Neurological examination showed normal muscle strength and skin sensation. Routine blood and biochemical tests, routine urine tests, liver function tests, kidney function tests, chest X-rays, electrocardiograms, echocardiography, Brain natriuretic peptide (BNP), troponin, and autonomic testings (Supine and standing blood pressure and residual urine ultrasound) did not reveal significant abnormities. Serum and urine immunofixation electrophoresis and serum-free light chain analysis did not detect any monoclonal proteins. The proband also had four affected older sisters: III-5, III-7, III-9, and III-11 (Table 1). All of them had vision loss and had undergone vitrectomy for both eyes. They all had blurred vision. According to these abnormalities, ATTRv amyloidosis was suspected; thus, this family underwent genetic testing of the *TTR* gene.

**TABLE 1:** Clinical phenotype in each affected subjects.

Number	Gender	c.307G>C	Onset age	Operation	Age of operation
I-2	F	—	50	NO	—
II-1	M	—	60	NO	—
II-4	F	—	48	NO	—
III-5	F	—	51	YRS	54
III-7	F	—	39	YES	42
III-9	F	+	41	YES	43
III-11	F	+	32	YES	43
III-13	F	+	31	YES	34
IV-7	F	+	unknown	NO	—
IV-9	F	+	unknown	NO	—

### Genetic analysis

**FIGURE 3:**
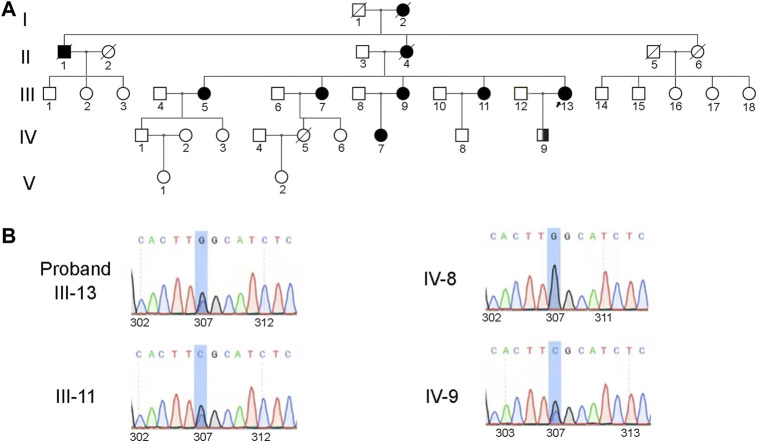
Heterozygous mutation (c.307G>C, p. G103R) in the TTR gene was identified in the family. **(A)** Pedigree of the family. Square symbols denote males, circle symbols denote females, solid symbols indicate the affected, open symbols indicate the unaffected, slash symbols indicate the deceased, symbol enclosing a vertical bar indicate the asymptomatic carrier, and an arrow below the symbol indicates the proband. **(B)** The results of family separation and analysis showed that the proband (IV-13), her daughter (IV-9), and her fourth sister (III-11) carried heterozygosity. The son of her fourth sister (IV-8) carried the wild-type gene.

Peripheral blood samples of the proband (IV-13), her daughter (IV-9), her fourth sister (III-11), and the son of her fourth sister (IV-8) were obtained for further genetic testing (Figure 3A). Sanger sequencing of the *TTR* gene revealed a heterozygous mutation c.307G>C (p.G103R) in the proband (IV-13), her daughter (IV-9), and her fourth sister (III-11) (Figure 3B). The clinical phenotype of her daughter (26 years old) was normal, while the proband and her fourth sister had low vision. Her daughter (IV-9) was an asymptomatic carrier. This mutation has been reported to be pathogenic and related to ATTRv amyloidosis (Chen et al., 2011; Xie et al., 2012; Yuan et al., 2012; Zhang et al., 2013; Liu et al., 2014; Yin et al., 2014; Xie et al., 2017; Li et al., 2021). Based on the clinical manifestation and genetic testing, the patients were diagnosed with ATTRv amyloidosis.

## Discussion

This study described a Chinese family with rare hereditary ocular amyloidosis in which vitreous hemorrhage was found in one eye. Sequencing revealed a heterozygous change in nucleotide c.307G>C (p.G103R) in exon 3 of the *TTR* gene in the proband (IV-13), her daughter (IV-9), and her fourth sister (III-11). The proband and her fourth sister had a similar clinical phenotype. The proband was characterized as having low vision, the presence of vitreous hemorrhage in the right eye**,** and multiple flocculent degenerations in the vitreous of the left eye. She had undergone multiple eye surgeries. Her daughter (IV-9), who carried heterozygosity, had no clinical phenotype. Based on the clinical phenotype and gene variation pattern, the patients were diagnosed with ATTRv amyloidosis. However, Congo red staining for biopsy samples was unavailable, and this was a limitation of our workup.

ATTRv amyloidosis is a rare multisystem disease that mainly involves the heart and the peripheral nervous system. It is a global disease that was first found in Portugal, Japan, and Sweden (Schmidt et al., 2017). Due to its point mutation, the *TTR* gene has an autosomal-dominant mode of transmission pattern. It is caused by the *TTR* protein, which forms amyloid fibers through misfolding and aggregation in organs and tissues. The *TTR* gene, which is located on chromosome 18, is small and has only four exons. More than 130 mutations have been explored through the *TTR* gene, most of which are pathogenic and amyloidogenic, leading to neuropathy, cardiomyopathy, and, even more uncommonly, ocular disease (Adams et al., 2021). No more than 10% of patients with ATTRv have ocular involvement (Minnella et al., 2021). The time from the onset of symptoms to the diagnosis of ATTRv amyloidosis can be as long as six years because of diverse clinical manifestations (Minnella et al., 2021). The *TTR* p.V50M mutation is the most common mutation globally. *TTR* is mainly synthesized in the liver, while the rest is synthesized by retinal pigment epithelium (RPE) cells and choroid plexuses of the brain. Liver transplantation is a treatment for neuropathy but not for cardiomyopathy and ocular disease (Planté-Bordeneuve and Said, 2011).

The clinical manifestations of ocular amyloidosis are abnormal conjunctival vessels, pupillary changes, keratoconjunctivitis sicca, lens capsule opacity, vitreous opacity, and secondary glaucoma (Ando et al., 1997; Choi et al., 2022). Among these, vitreous opacity is the most common ocular presentation. The symptoms of vitreous opacity are floating objects in front of the eyes and progressive vision loss. The vitreous showed white flocculent opacity. Hara et al. reported that 36% of p.V50M patients developed vitreous opacities, while 18% developed glaucoma, even after liver transplantation. This study also suggested that *TTR* is synthesized by RPE, not the liver, in hereditary vitreous amyloidosis (Hara and Ryuhei, 2010). A Chinese study reported that in a p.R54G family, 22% developed vitreous opacities, with the age of disease onset being female >55 years old and male >40 years old (Yining et al., 2011). Haraoka et al. found amyloidosis deposits in the inner retinal layers rather than close to RPE (Haraoka et al., 2002). 0″Hearn et al. reported that two patients with p.E74G mutations, accompanied by an increased intravitreal concentration of VEGF, suggested posterior segment ischemia (O"Hearn et al., 2007).

To our knowledge, however, the current study presents the first cases of preretinal neovascularization associated with the p.G103R mutation in ATTRv amyloidosis. Vitreous hemorrhage was seen in our proband, and VEGF, BFGF, IL-6, IL-10, VCAM, and IL-8 concentrations were increased significantly. Our results suggest that not only ischemia but also inflammation occurred in hereditary vitreous amyloidosis with vitreous hemorrhage. During the operation, vitreous hemorrhage, retinal vein occlusion, and retinal holes in the peripheral retina were found. We suspect vitreous hemorrhage in the right eye may be caused by amyloid deposition on the retinal vascular wall, which obstructs oxygen delivery in the surrounding tissues and induces the upregulation of the vascular endothelial growth factor after hypoxia. It may also be secondary to damage that amyloid infiltration causes to the vascular wall. Combined cataract surgery can damage the blood–aqueous barrier even more severely. Recurrent retinal hemorrhage causes inflammation and edema in the eye, causing a cascade of inflammation. No combined operation was performed on the left eye, the damage to the blood–aqueous barrier was not serious, and the damage to the vascular wall of the left eye was not obvious. Retinal and choroidal amyloid deposition occurs in the last stage of the disease (Kakihara et al., 2022). Large deposits of amyloid were seen in the anterior and subretinal areas of our patient. These deposits might play a role in the development of retinal vein occlusion and retinal hemorrhage. Rousseau et al. reported that patients with p.V50M mutations of vitreous amyloidosis had choroidal amyloid angiopathy in late indocyanine green angiography (ICGA) (Antoine et al., 2018). Abnormal choroidal vessels might be a direct infiltration of amyloids. Therefore, we suggest conducting ICGA examination before the operation to evaluate choroidal amyloid angiopathy, which is a risk factor for surgery.

Another important ocular manifestation is secondary glaucoma. The p.V50M mutation has been reported to be associated with anterior segment neovascularization and neovascular glaucoma (Antoine et al., 2018). The deposition of amyloid protein around blood vessels in the conjunctiva and sclera can lead to an increase in scleral venous pressure and intraocular pressure; ocular amyloid deposition blocks the trabecular meshwork and Schlemm canal, and the discharge of aqueous humor is blocked, resulting in glaucoma (Minnella et al., 2021). Kimura et al. reported that the average incidence rate of secondary glaucoma was 24%, and the incidence of glaucoma in patients with the p.V50M mutation was 17%, which was significantly lower than that of other mutation types, for example, p.Y134C and p. S70I (Kimura et al., 2003). A study of 79 eyes of 42 patients with hereditary vitreous amyloidosis found that those who had undergone PPV had a higher incidence of glaucoma than those who had not (56.1% vs. 10.5%) (Beiro et al., 2012). In our study, the proband had undergone multiple PPV, and the IOP was higher than normal.Choi et al. (2022) reported that in patients with p. D58A and p.T79K mutations, anterior lens capsule opacity and retinal deposits were observed. Posterior subcapsular lens opacity was observed in our patient. In addition, typical clinical manifestations of ocular amyloidosis, including abnormal conjunctival vessels, pupillary changes, and keratoconjunctivitis sicca, were not observed in this patient.

Most ocular amyloidosis cases are caused by a p.V50M mutation of *TTR*, and only a few rare mutations have been reported. This study demonstrated a p.G103R mutation in the *TTR* gene of a proband from a Han Chinese family. Coincidentally, this mutation has been previously reported in Chinese families (Chen et al., 2011; Xie et al., 2012; Yuan et al., 2012; Zhang et al., 2013; Liu et al., 2014; Yin et al., 2014; Xie et al., 2017; Li et al., 2021). Like our study, there were no systemic symptoms except for ocular amyloidosis in all family members at its early stage. To the best of our knowledge, there have been no previous reports of vitreous hemorrhage in ocular amyloidosis caused by the p.G103R mutation. In our study, we summarized the clinical manifestations of the p.G103R mutation of the *TTR* gene. They indicate that p.G103R variation is a type of *TTR* mutation specific to the Chinese population. We suggest screening patients in China for this variation in *TTR*. The p.K55T and p.L75R mutations of *TTR* genes have also been reported in Chinese families (Long et al., 2012). Recently, a Chinese study reported that the *TTR* p.K55N mutation presented with sensorimotor neuropathy and severe autonomic dysfunction four years after the first diagnosis of ocular amyloidosis (Xu et al., 2021). Most patients with p. G103R have only been reported to have ocular symptoms. Yet extraocular symptoms of the p. G103R mutation were reported in Li et al. (2021)’s study, which involved three families with six patients and two asymptomatic carriers. All the patients started with blurred vision and vitreous amyloidosis, and most of them developed sensory-motor polyneuropathies several years later. Liu et al. (2014) reported that patients with the p.G103R mutation also had mild polyneuropathy and cardiac amyloidosis in addition to ocular manifestations. TTR stabilizer tafamidis has been shown to slow the deterioration of neurological function of ATTRv amyloidosis, but has no effect on ocular symptoms, possibly because it cannot cross the blood-brain and blood-retinal barriers (Salvi et al., 2018). The proband in our study is a 48-year-old woman who has no systemic complications of ATTRv amyloidosis. We will continue a long-term follow-up study.

## Materials and methods

### Participants and clinical data

All research involved in this study adhered to the tenets of the Declaration of Helsinki and was approved by the Institutional Review Board of Second Affiliated Hospital of Zhejiang University. Informed written consent was obtained from all participating individuals in this study.

Ophthalmological examinations including visual acuity test, slit-lamp photograph, B-mode ultrasonography, wide-field retinal imaging (Optos 200Tx,Marlborough, MA, United States), optical coherence tomography (OCT) (Heidelberg HRT II, Heidelberg, Germany), intraocular pressure were performed. Routine blood tests and biochemical tests, urine routine examination, liver function tests, kidney function tests, BNP, myocardial enzymes, chest X-rays, electrocardiograph, echocardiography, residual urine ultrasound and neurologic examinations were performed to reveal potential dysfunction of these organs. Vitreous fluid of the proband were collected during vitrectomy and sent out for analysis (Flow Cytometry Analysis, Beijing Giantmed Medical Diagnostics Lab).

### Genetic analysis

Peripheral blood sample of the proband was obtained for further Sanger sequencing of *TTR* gene. Results were mapped in respect to the *TTR* reference sequence (Ensembl Gene ID: ENSG00000118271). The variants were further validated by Sanger sequencing in all available family members. The nomenclature used standard numbering beginning at the Met initiation codon, which had a 20-amino-acid difference with historic nomenclature reported in older literature (e.g., p.G103R would be referred to as p.G83R).

## Data Availability

The datasets presented in this study can be found in online repositories. The names of the repository/repositories and accession number(s) can be found in the article/Supplementary Material.
