# Supplementary figures and images for: Case Report: Hereditary transthyretin (ATTRv) amyloidosis: The p.G103R mutation of the transthyretin gene in a Han Chinese family is associated with vitreous hemorrhage
Source: Front Genet. 2022 Sep 15;13:972501. doi: 10.3389/fgene.2022.972501 (PMC9520364; doi:10.3389/fgene.2022.972501)

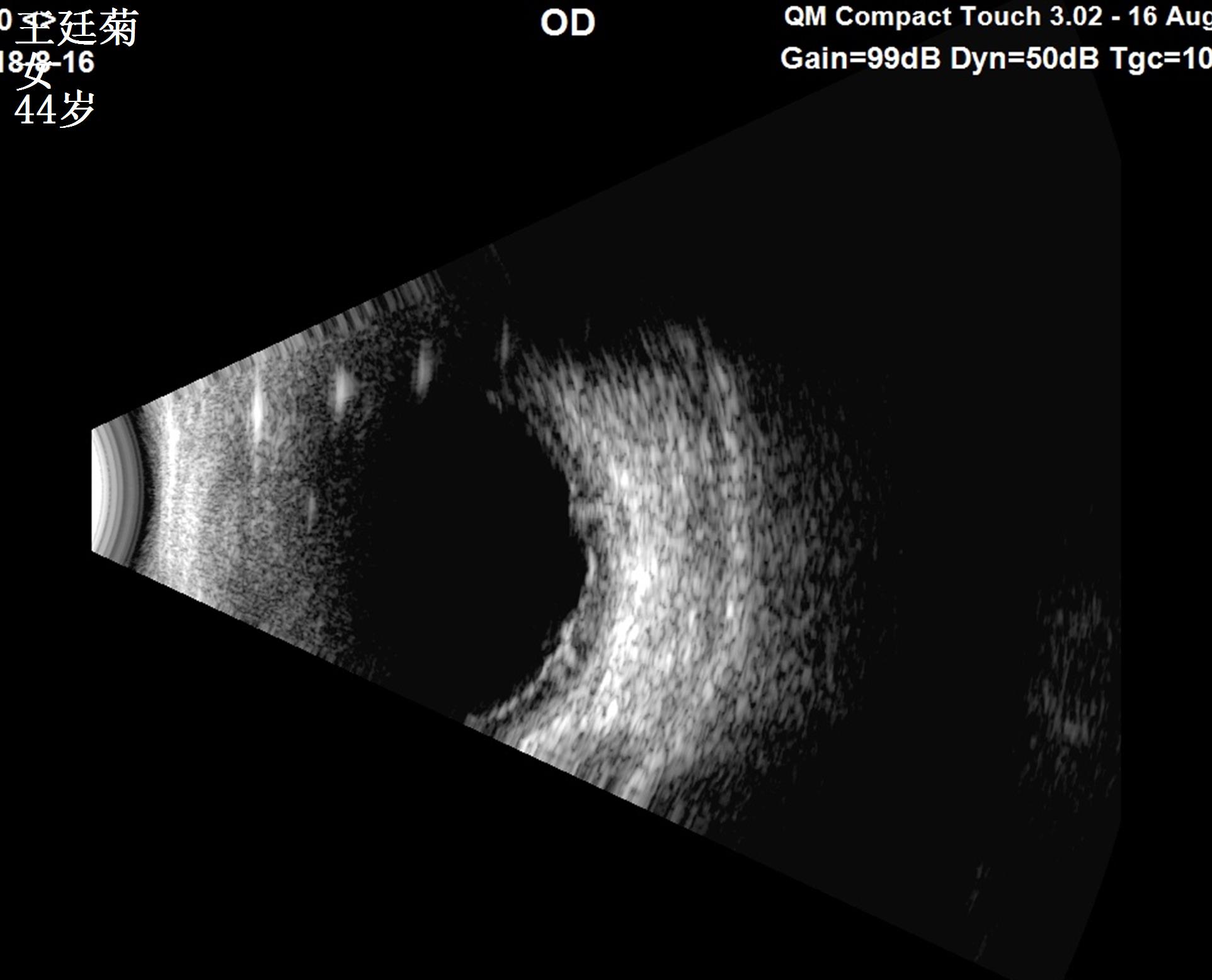

Supplement: Supplementary file 1 [file Image15.JPEG]

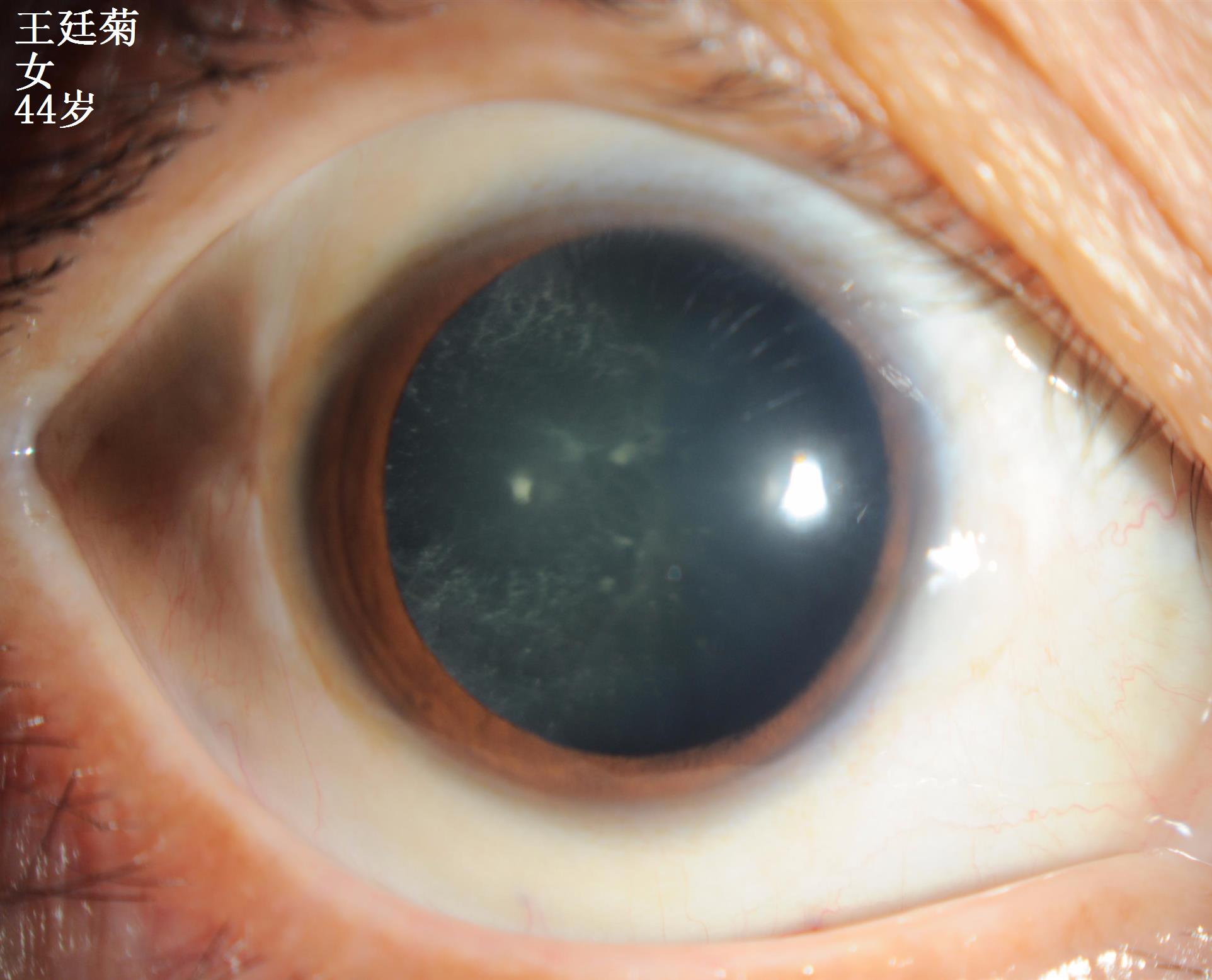

Supplement: Supplementary file 2 [file Image3.JPEG]

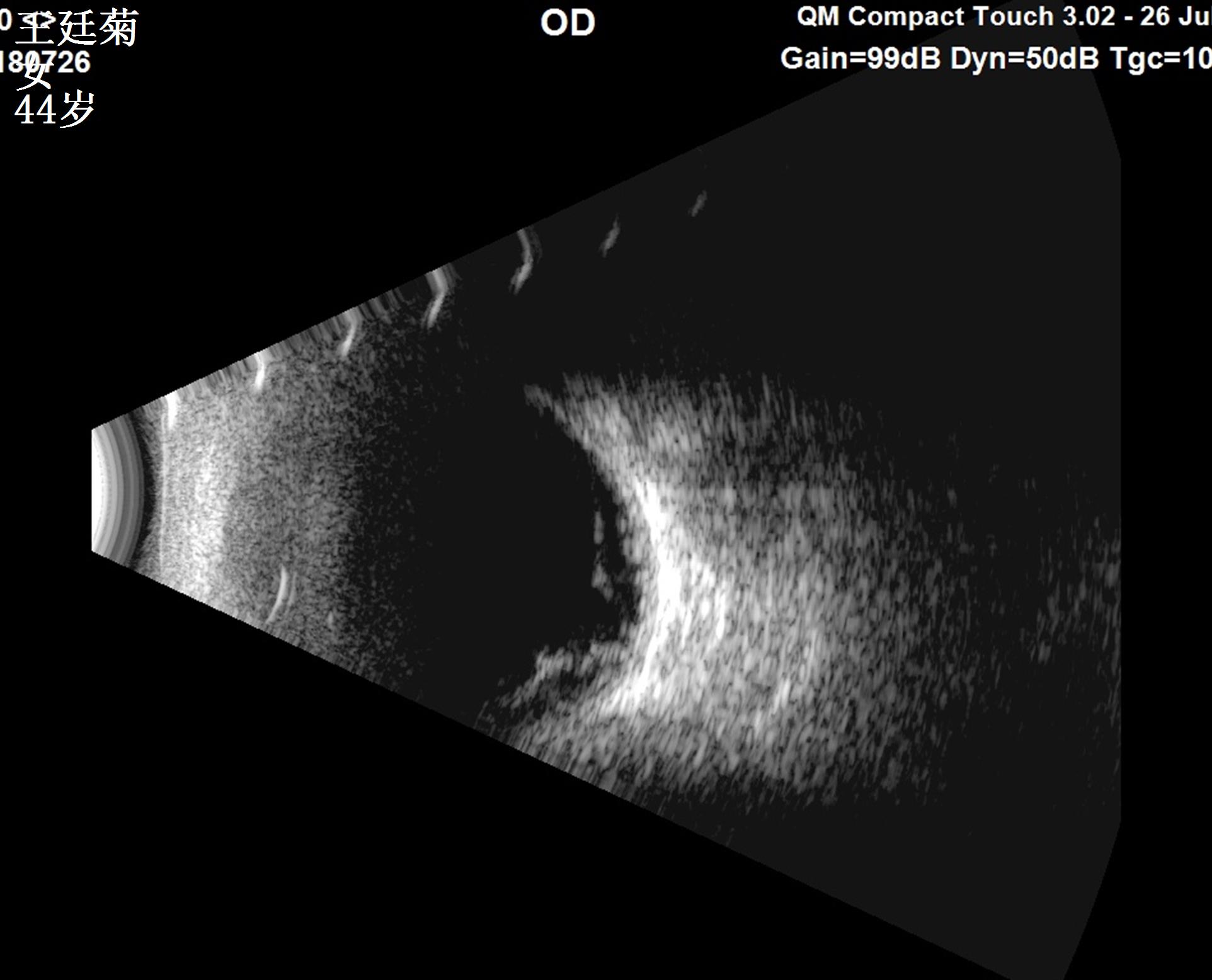

Supplement: Supplementary file 3 [file Image9.JPEG]

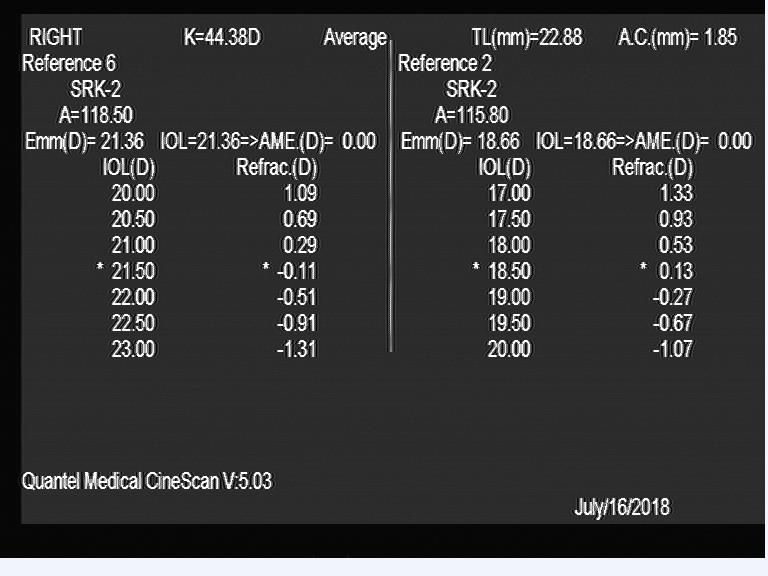

Supplement: Supplementary file 4 [file Image1.JPEG]

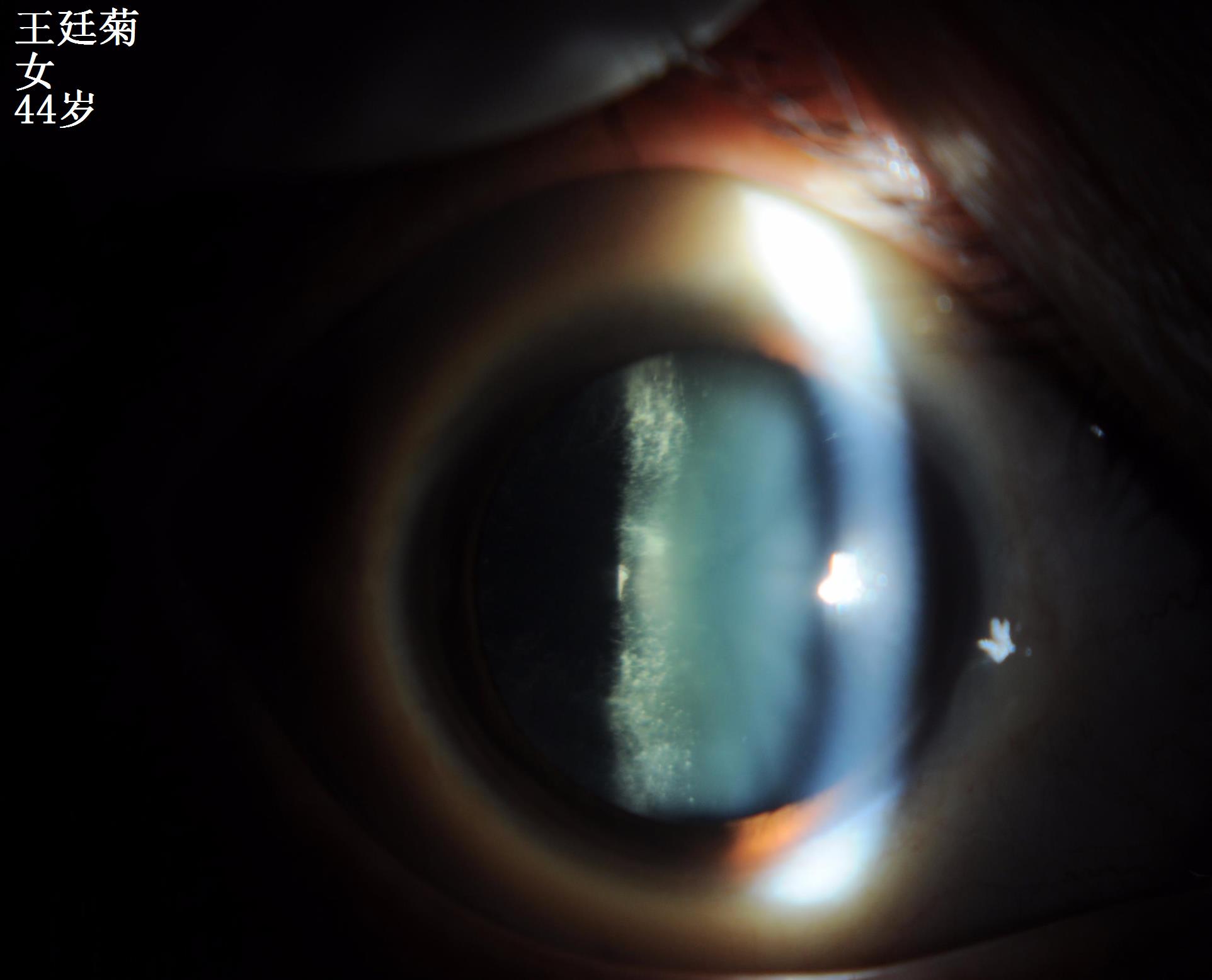

Supplement: Supplementary file 5 [file Image4.JPEG]

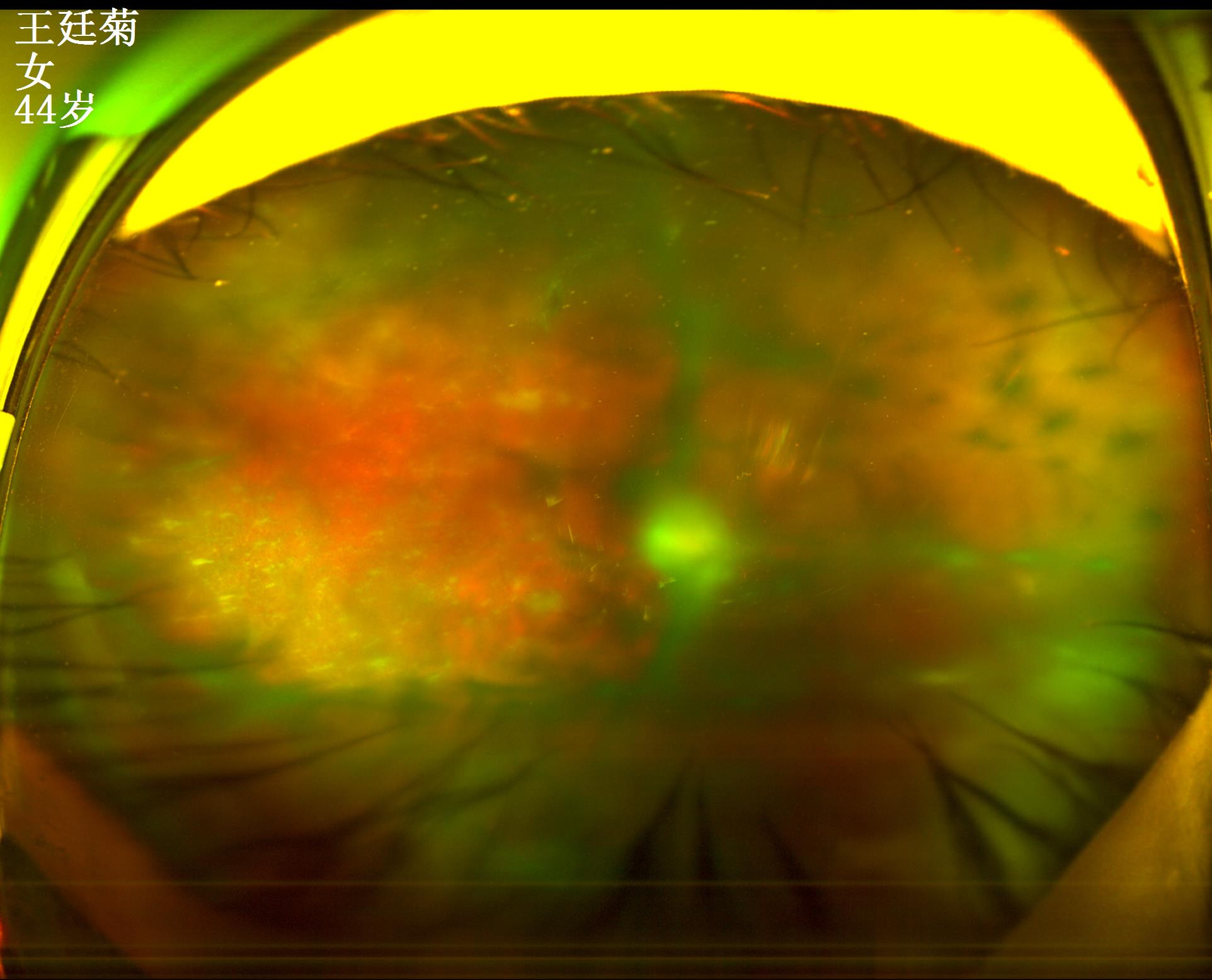

Supplement: Supplementary file 6 [file Image7.JPEG]

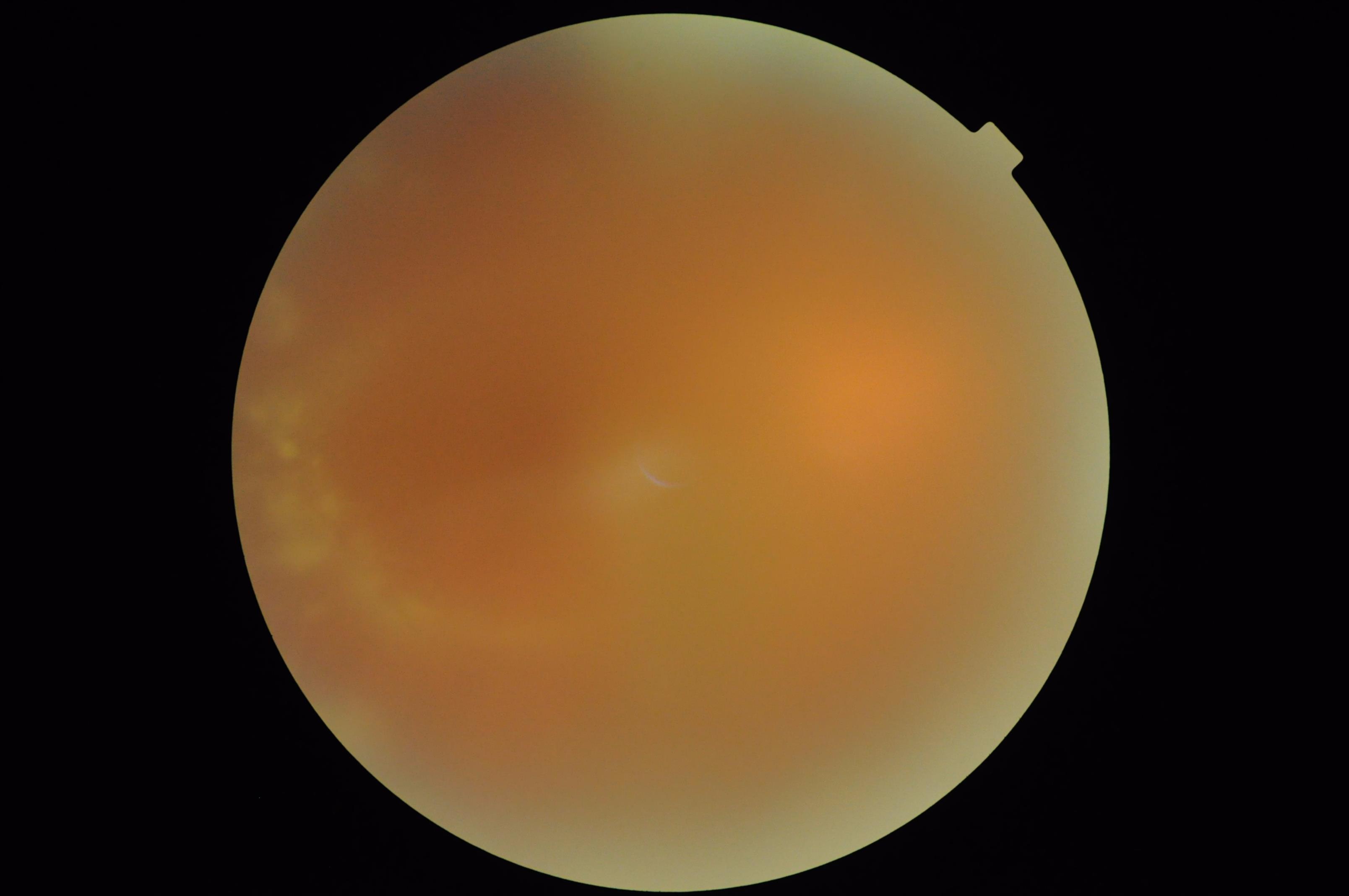

Supplement: Supplementary file 7 [file Image2.JPEG]

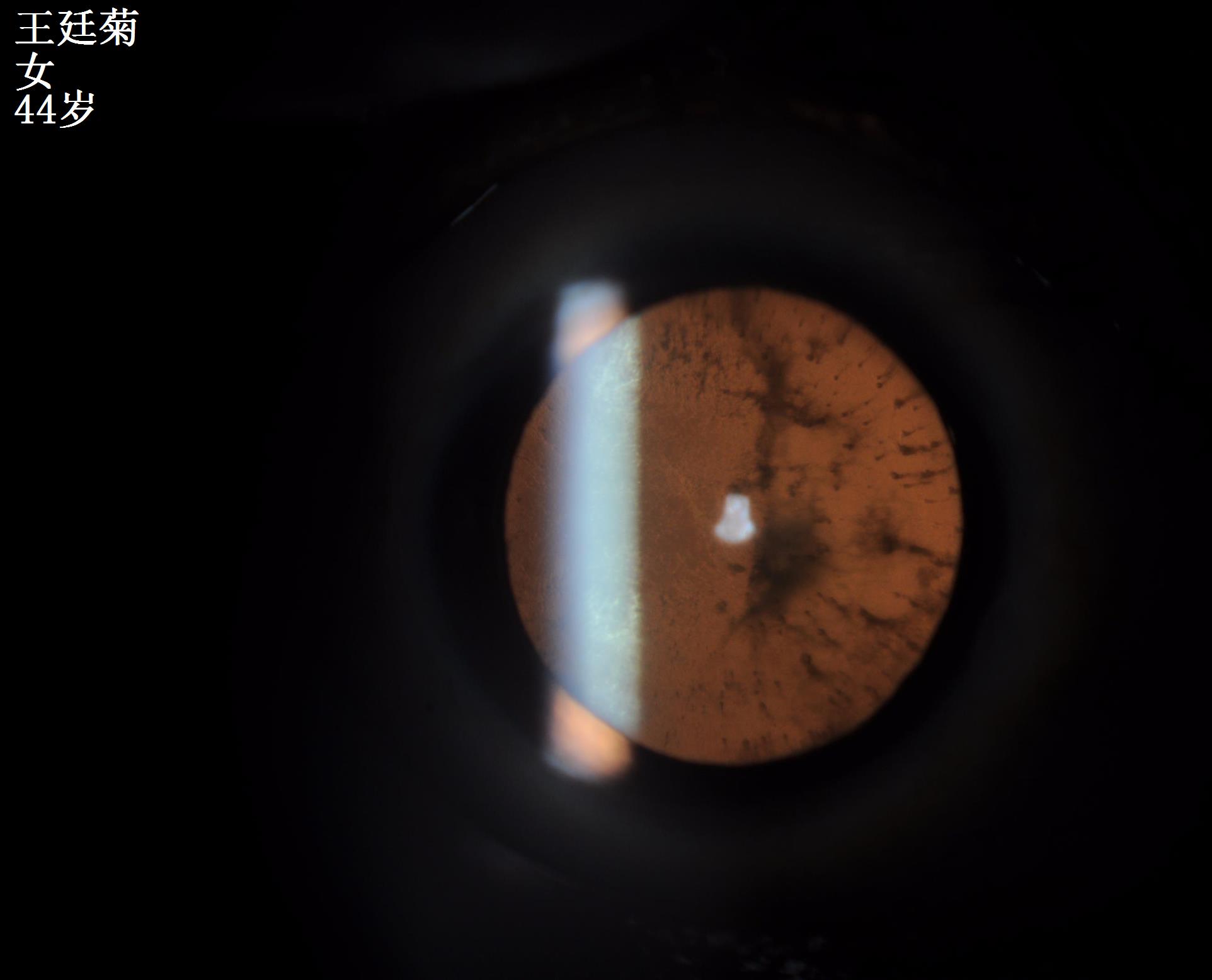

Supplement: Supplementary file 8 [file Image5.JPEG]

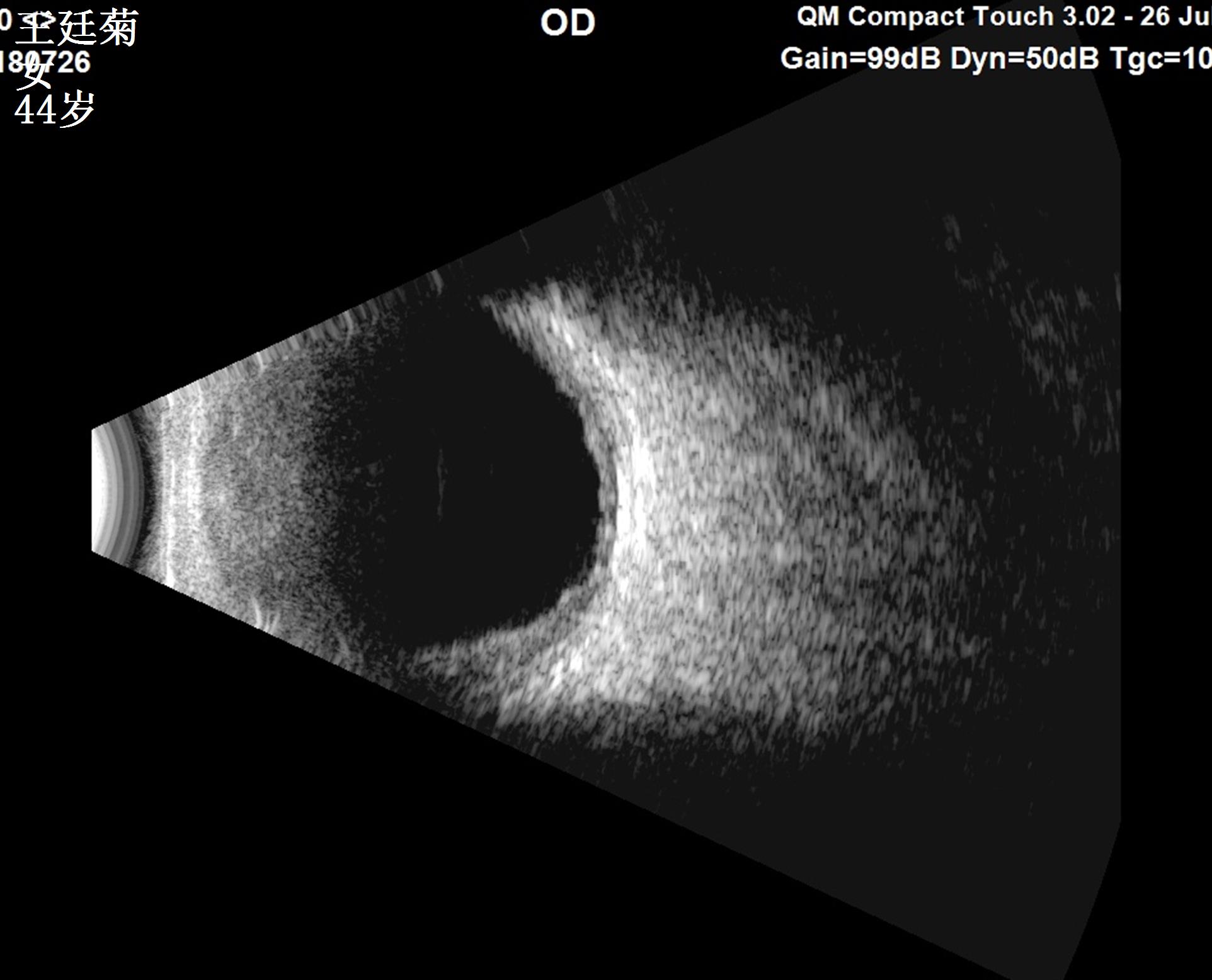

Supplement: Supplementary file 9 [file Image10.JPEG]

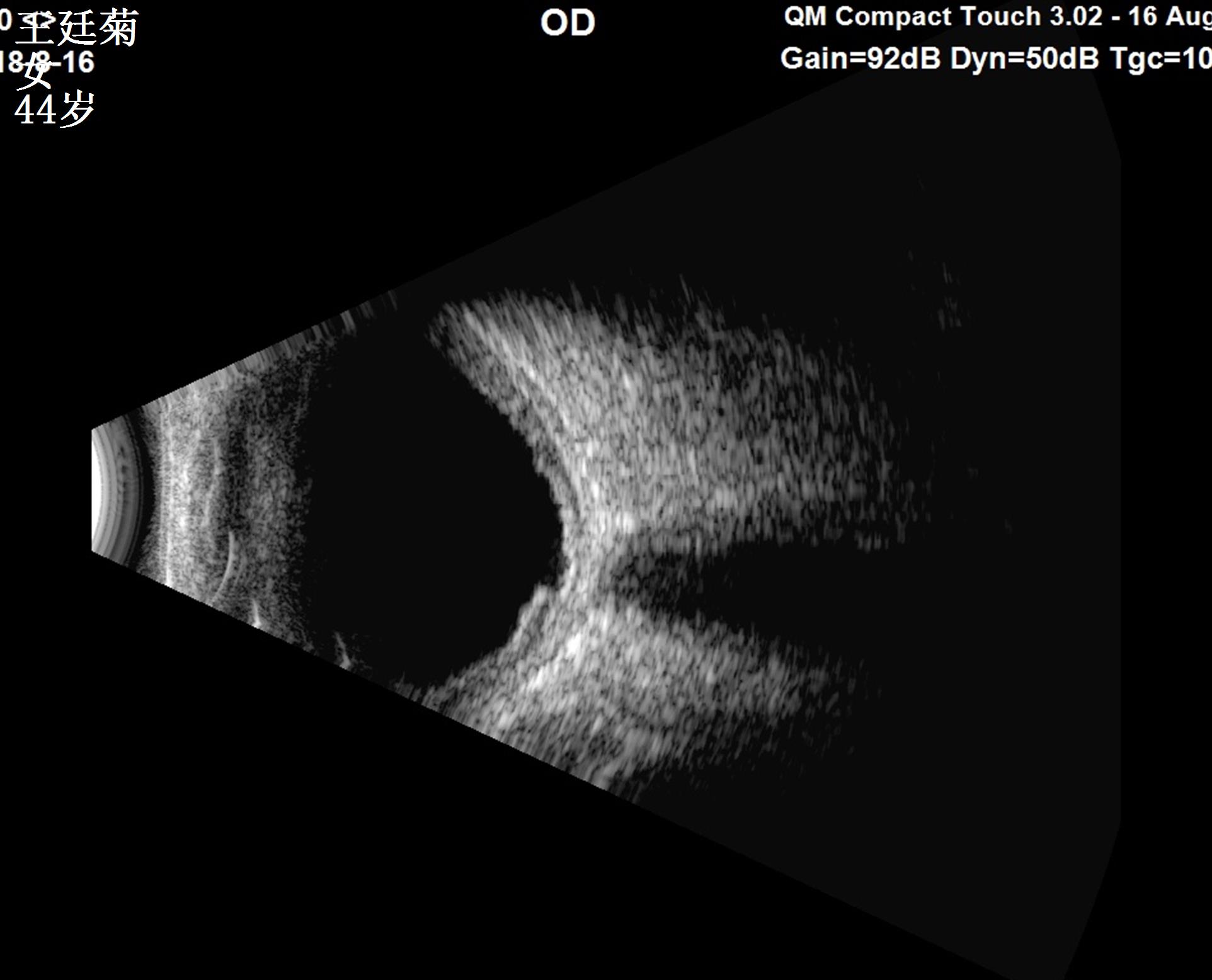

Supplement: Supplementary file 10 [file Image14.JPEG]

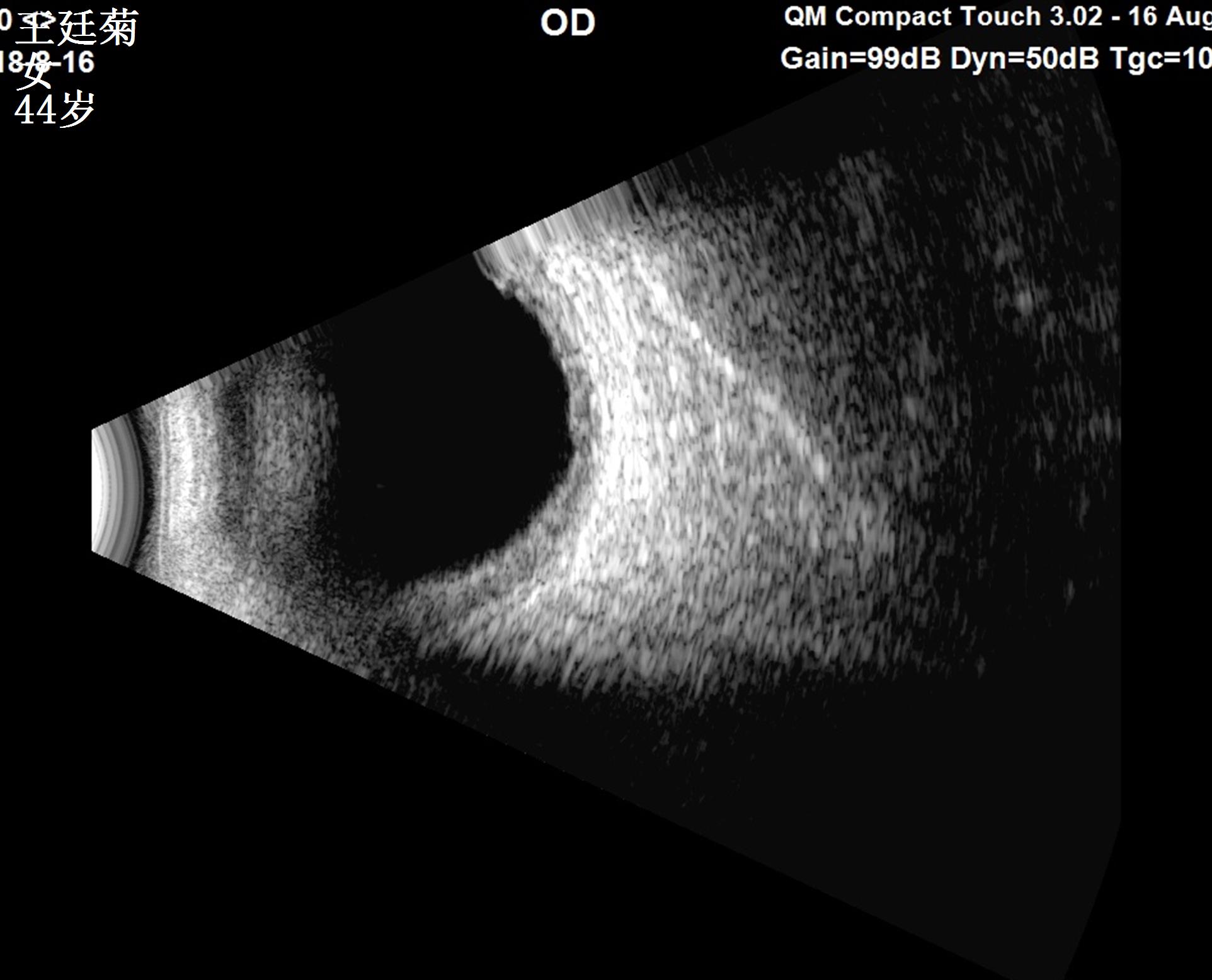

Supplement: Supplementary file 11 [file Image12.JPEG]

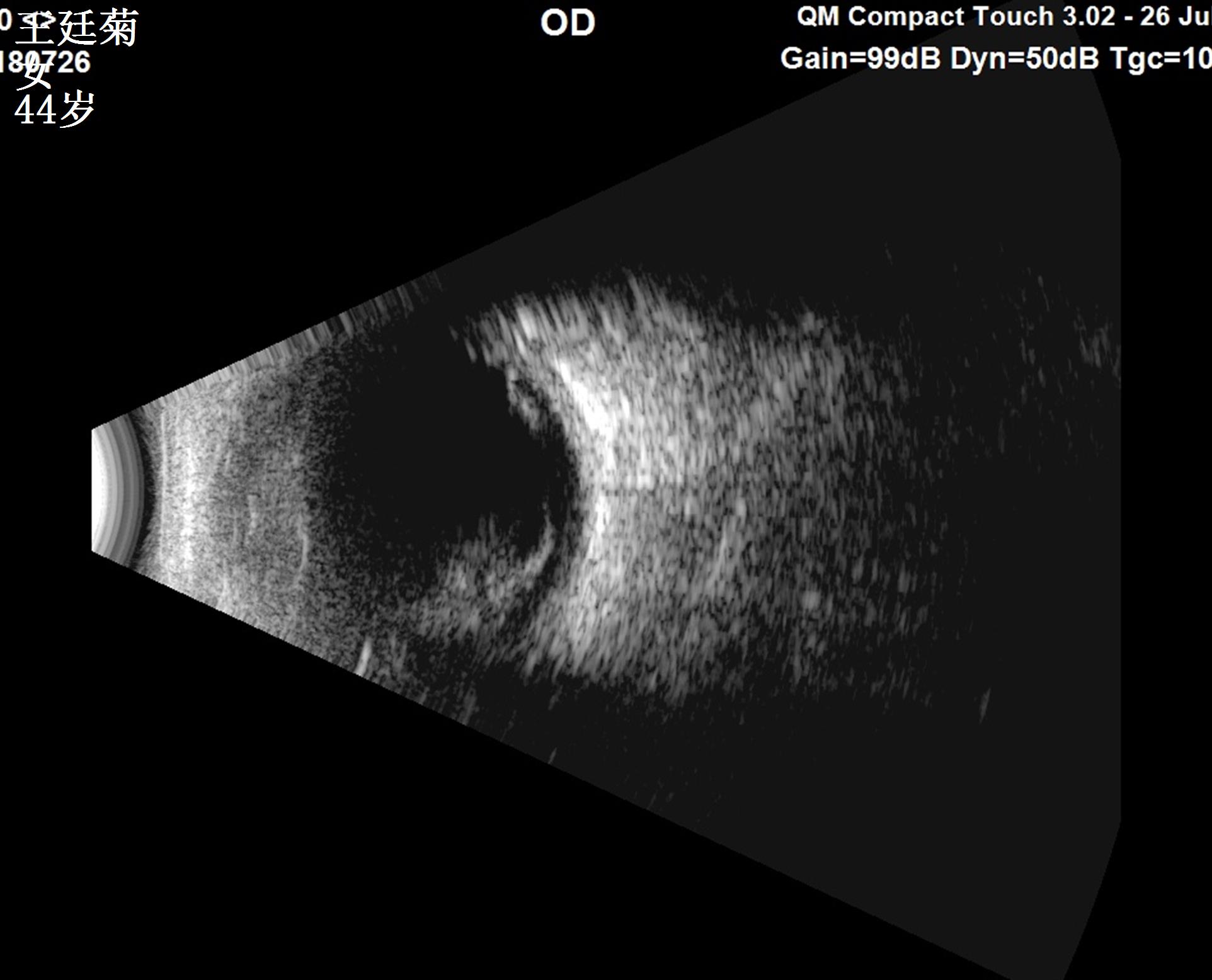

Supplement: Supplementary file 12 [file Image11.JPEG]

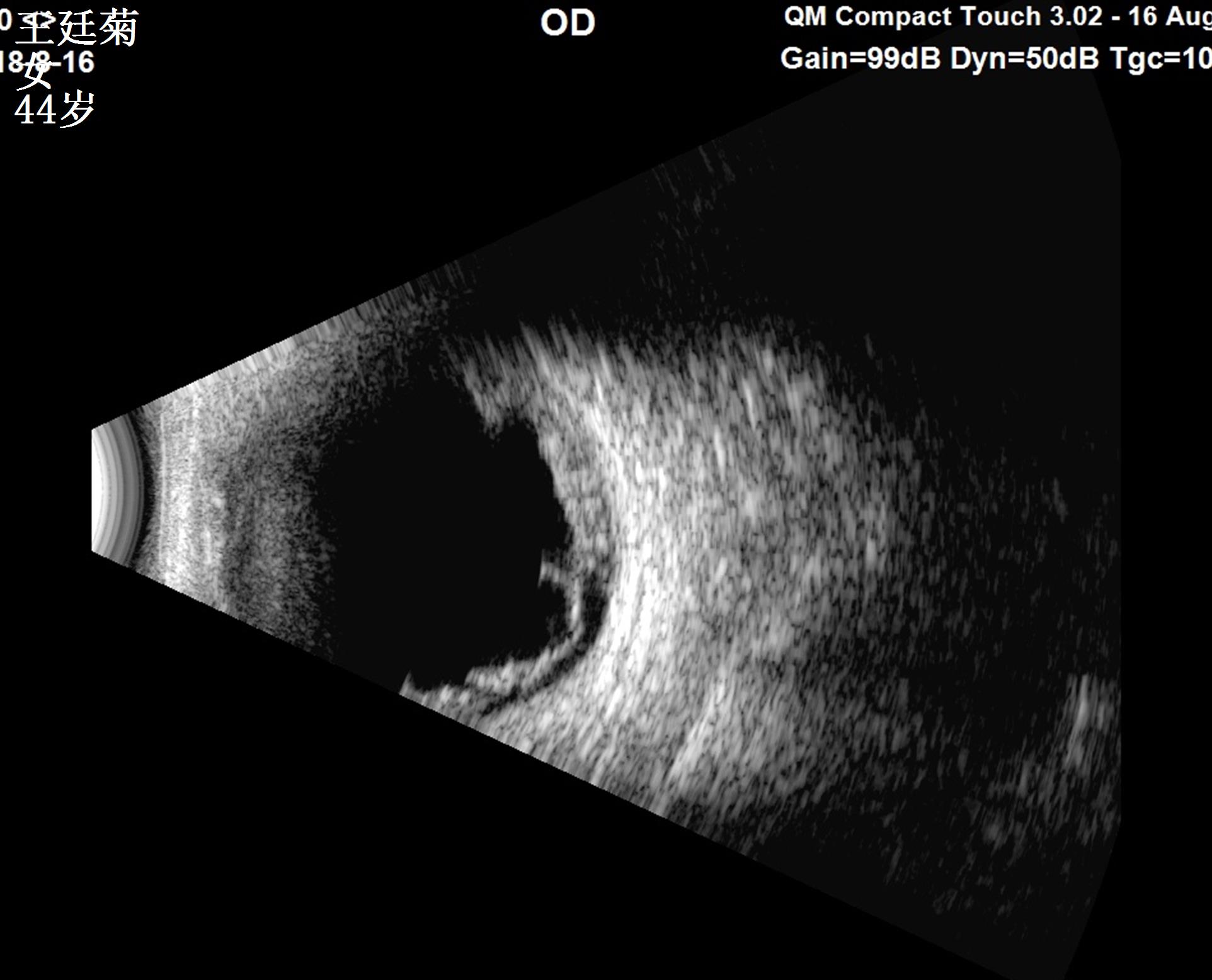

Supplement: Supplementary file 13 [file Image13.JPEG]

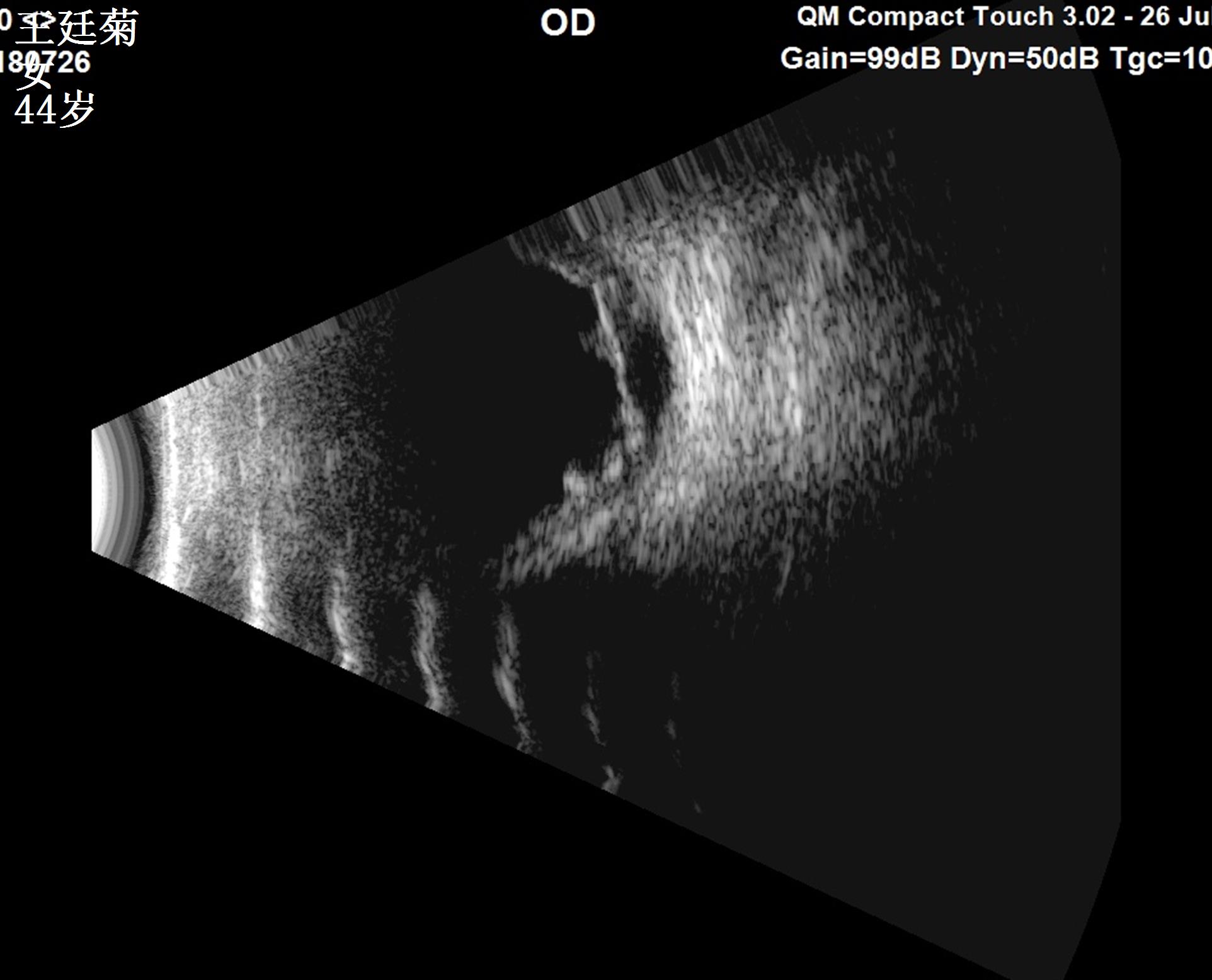

Supplement: Supplementary file 14 [file Image8.JPEG]

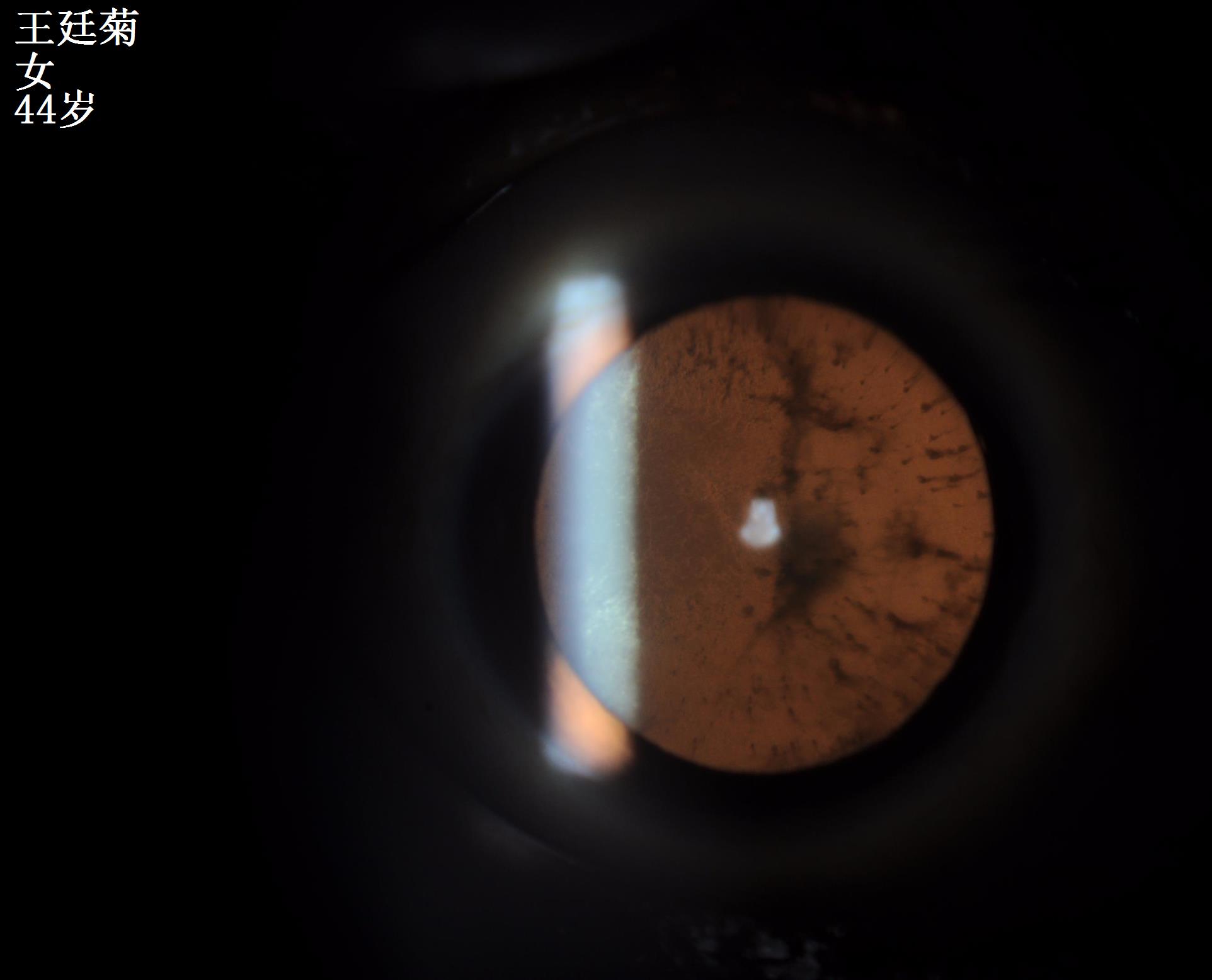

Supplement: Supplementary file 15 [file Image6.JPEG]
